# Health outcomes, education, healthcare delivery and quality – 3037. Allergy immunotherapy quality of life and perceived efficacy opinion survey

**DOI:** 10.1186/1939-4551-6-S1-P211

**Published:** 2013-04-23

**Authors:** Praveen Buddiga, Malik Baz

**Affiliations:** 1 University of California, San Francisco, Baz Allergy, Asthma & Sinus Center, Fresno, CA, USA

## Background

The effectiveness of Allergy Immunotherapy is known to subjectively improve quality of life. An opinion survey was designed to elicit the perceived efficacy among immunotherapy patients with regards to the mitigation of symptoms of Allergic rhinitis, Allergic conjunctivitis, Atopic dermatitis and improvement in Asthma control.

## Methods

A 10 question voluntary anonymous survey on paper was designed by a two-allergist team to assess the patient perception of the improvement in quality of life on Allergy Immunotherapy by the subcutaneous route. The measures that were tested was perceived reduction as well as improved control with regards to disease states such as Allergic rhinitis, Asthma, Allergic conjunctivitis and Atopic dermatitis. Distribution of the voluntary survey was undertaken at 8 different sites. Responses were accepted over a testing period of 14 working days from May 1 - May 18, 2012.

## Results

Of the 500 respondents from 8 different sites, 463 completed the complete survey. Only the data from completed surveys underwent statistical analysis. 463 respondents were in the age range of 5 years to 84 years and consisted of 44% male and 56 % female. The data analyzed corresponded to 1489 years (one thousand four hundred eighty nine years) of subcutaneous injections. 82 % of the respondents expressed the alleviation of symptoms of allergic rhinitis, 43 % improvement in asthma control, 55 % decrease in recurrent sinusitis, 56% decrease in allergic conjunctivitis and 18 % decrease in atopic dermatitis. 97.6 % of respondents reported an improvement in quality of life, 1.5 % reported no improvement and 0.9 % were unsure.

## Conclusions

Allergists caring for patients with atopic conditions can be reassured that the perceived clinical efficacy of subcutaneous allergy immunotherapy is at an extremely high level with regards to resolution of symptoms within the atopic disease states that it is indicated for. Opinion surveys can be a very helpful tool in assessing current patient level of satisfaction to therapy regarding atopic disease states.

